# *Wyeomyia confusa Lispivirus* (WcLispV-SP): a novel neotropical mosquito virus in the *Lispiviridae* family

**DOI:** 10.1007/s00705-026-06646-w

**Published:** 2026-05-15

**Authors:** Lilian de Oliveira Guimarães, Roseane da Silva Couto, Simone Luchetta Reginato, Luis Filipe Mucci, Ramendra Pati Pandey, Vera Lucia Fonseca de Camargo-Neves, Antonio Charlys da Costa, Karin Kirchgatter, Elcio Leal

**Affiliations:** 1 Pasteur Institute, São Paulo, SP 01027-000 Brazil; 2 Viral Diversity Laboratory, Institute of Biological Sciences, Federal University of Pará, Belém, Pará Brazil; 3 Institute of Biosciences and Bioengineering, D. Y. Patil International University, Pune, Maharashtra India; 4 Institute of Tropical Medicine, Faculty of Medicine, University of São Paulo, São Paulo, SP 05403-000 Brazil

## Abstract

Metatranscriptomic analysis of *Wyeomyia confusa* mosquitoes collected in the Atlantic Forest (Pindamonhangaba, São Paulo, Brazil) led to the identification of a previously uncharacterized virus, designated *Wyeomyia confusa Lispivirus* (WcLispV-SP), classified within the family *Lispiviridae*, genus *Canmovirus*. The viral genome consists of a negative-sense single-stranded RNA (ssRNA−) of 12,698 nucleotides, encoding six open reading frames (ORFs): nucleoprotein (N), two hypothetical proteins (HP/1 and HP/2), glycoprotein (G), ORFan protein, and RNA-dependent RNA polymerase (RdRp-L). Phylogenetic analysis supports the classification of WcLispV-SP as a distinct species within the genus *Canmovirus*. Structural analysis of the RdRp revealed conserved domains and catalytic motifs characteristic of members of the order *Mononegavirales*, supporting its functional integrity. These findings expand the known diversity of the *Lispiviridae* family and highlight the utility of metagenomic approaches for the discovery and characterization of RNA viruses associated with Neotropical sylvatic mosquitoes.

## Introduction

The mosquito *Wyeomyia confusa* belongs to the family *Culicidae* and the genus *Wyeomyia*. This species is typically associated with wild environments and is found in humid tropical ecosystems of the Neotropical region (such as the Amazon Rainforest), including areas of the Atlantic Forest like the municipality of Pindamonhangaba (São Paulo), where it occurs in shaded, high-humidity habitats. Immature stages develop predominantly in phytotelmata, such as bromeliads, which provide ideal conditions of moisture, organic matter, and protection from predators for larval development [1].

Species of the genus *Wyeomyia* are known for maintaining a close association with forested environments, exhibiting predominantly zoophilic behavior, with feeding preferences directed toward small vertebrates [2]. Although species of this genus are not among the main vectors of arboviruses affecting humans, the Zika virus has been detected in specimens of *Wyeomyia confusa* in the Atlantic Forest, in the municipality of São Paulo [3]. Furthermore, recent studies have shown that *Wyeomyia* can act as a host or reservoir for poorly characterized viruses, particularly those detected through metagenomic approaches in highly biodiversity ecosystems [4, 5].

The characterization of novel viruses in wild insects is a crucial step toward understanding global viral diversity and anticipating potential spillover events [6, 7]. Investigating previously undescribed viruses enables the identification of emerging lineages and provides insights into the evolutionary and ecological mechanisms that facilitate their adaptation to new hosts.

Because of the history of arboviral outbreaks that have affected public health worldwide, the characterization of viruses linked with mosquitoes is very important. The advent of Zika and chikungunya viruses, as well as recurrent dengue and yellow fever epidemics, highlights these arthropods’ ability to spread emerging infectious diseases [8–11]. These incidents highlight how crucial it is to conduct ongoing viral monitoring, characterize the genome, and identify new viruses that are circulating in mosquito populations early on in order to reduce epidemiological risks and aid in preventative measures.

In this context, the study of viruses associated with *Wyeomyia confusa* contributes to expanding our understanding of viral diversity in natural environments and the potential mechanisms involved in the maintenance and evolution of emerging viruses. The identification and genomic characterization of *Wyeomyia confusa Lispivirus* (WcLispV-SP) help fill gaps in our knowledge of the diversity and phylogeny of viruses within the *Lispiviridae* family, providing valuable insights for evolutionary, taxonomic, and environmental surveillance studies.

This study used phylogenetic analysis, genetic distance metrics (SDTv), and structural conservation of RdRp catalytic motifs to identify, molecularly characterize, and evaluate the taxonomic position of *Wyeomyia confusa Lispivirus* (WcLispV-SP), which was found in a metagenomic sample (Meta17). This method aims to advance the evolutionary knowledge of viruses linked to wild mosquitoes as well as viral surveillance.

## Materials and methods

### Sample collection and pool preparation for viral metagenomic screening

In a broader study, mosquitoes were collected from 2019 to 2020 in diverse habitats within nine municipalities from the State of São Paulo. Adult female specimens non-engorged were grouped according to collection date, location, and species identity, aiming to conduct a comprehensive viral metagenomic analysis. Here, we specifically analyzed in detail the sample named Meta17, focusing on the identification and characterization of a novel mosquito virus in the *Lispiviridae* family.

Meta17 is composed by 44 specimens of *Wyeomyia confusa* mosquitoes that were collected (by hand net and Shannon trap) at ground level in a forested area within the Parque Natural Municipal do Trabiju, Pindamonhangaba, São Paulo, Brazil (22°50’19.9"S, 45°31’24.7"W and 22°50’27.6"S, 45°31’20.2"W). These collections occurred on January 7, 2020, Pindamonhangaba (SP) experienced typical rainy summer conditions in the Vale do Paraíba region, with temperatures ranging from average lows of 18 °C to highs near 28 °C, high humidity (above 80%). Mosquitoes were grouped into a single metagenomic pool (Meta17), enhancing sensitivity for viral detection while minimizing handling bias.

### RNA extraction and sequencing procedures

Meta17 was homogenized using a FastPrep-96 system (MP Biomedicals, USA) at 1500 rpm for 1 min with 900 µL of Hanks’ Balanced Salt Solution (HBSS) and 1 g of ceramic beads (1.4 mm diameter). After homogenization, the sample was centrifuged at 14,000 rpm for 10 min, and 500 µL of the supernatant was filtered through 0.45 μm membranes (Merck Millipore, MA, USA) to remove debris and large particles.

Complementary DNA (cDNA) synthesis was carried out using SuperScript IV reverse transcriptase (Thermo Fisher Scientific), followed by second-strand synthesis with the DNA Polymerase I Large (Klenow) Fragment (Promega). The double-stranded DNA was then used for library construction using the Nextera XT DNA Library Preparation Kit (Illumina, USA). Sequencing was performed on an Illumina MiSeq platform at the Central Laboratory of the Hospital das Clínicas, University of São Paulo.

### Bioinformatics workflow

Raw reads from next-generation sequencing (NGS) were first evaluated using FastQC v0.12.1 to assess base quality, GC content, and read length distribution (accessed on September 23, 2025) [12]. Adapter sequences and low-quality regions were trimmed using Trimmomatic with the parameters LEADING:3, TRAILING:3, SLIDINGWINDOW:4:15, and MINLEN:36, using the TruSeq2-PE adapter set [13]. The quality of the processed reads was reassessed with FastQC to confirm data integrity [12].

Assembly of the viral genomes was conducted using rnaviralSPAdes v3.15.5 [14]. The viral genome corresponding to WcLispV-SP was assembled as a single continuous contig, without the need for manual joining or scaffolding of multiple contigs. Genome completeness was defined based on: (i) recovery of a single contiguous contig, (ii) presence of all expected ORFs characteristic of *Lispiviridae*, (iii) absence of internal gaps, and (iv) consistent read coverage across the genome, as supported by read mapping and depth analysis.

Genome termini (5′ and 3′ ends) were inferred based on comparison with related viruses and conserved genomic features of members of the order *Mononegavirales*; however, no experimental validation (e.g., RACE) was performed.

Assembly validation was performed by mapping raw reads back to the assembled contig. Reads obtained from metagenomic sequencing were mapped against the assembled viral genome using Bowtie2 in very-sensitive end-to-end mode with default parameters [15]. The resulting alignment files were processed using SAMtools to generate sorted BAM files [16]. Genome coverage and mapping statistics were subsequently assessed using Qualimap (BamQC module), including mean sequencing depth, coverage distribution, and total number of mapped bases [17]. Depth of coverage across the genome was additionally calculated using SAMtools depth [16] and summary statistics (e.g., mean coverage) were computed using Datamash.

Viral contigs were identified using DIAMOND v2.1.9 (BLASTx mode) against a curated viral protein database [18] derived from the Serratus RdRp database (https://serratus.io, accessed on September 2025). Hits were filtered based on e-value thresholds (< 1e − 5) and alignment scores, and sequence similarity metrics (including percentage identity and alignment coverage), as detailed in Table 1. Taxonomic classification was performed using Kraken2 configured with the prebuilt RefSeq Standard-Full database, which includes sequences from archaea, bacteria, viruses, plasmids, the human genome, and UniVec_Core [19].

Additional confirmation was performed using BLASTn and BLASTp searches against the NCBI GenBank database [20]. No host read depletion step or computational filtering against a host reference genome was performed; however, stringent filtering criteria and downstream validation steps were applied to minimize potential background noise. No experimental validation (e.g., PCR amplification or Sanger sequencing) was performed, and all analyses are based on in silico approaches.

We found one complete genome of a virus (named WcLispV-SP, GenBank PX992751) that belongs to the *Lispiviridae* family. For comparative purposes, we also used the reference sequences of this viral family, based on the International Committee on Taxonomy of Viruses (ICTV) (https://ictv.global/report/chapter/lispiviridae/lispiviridae, accessed on September 23, 2025) [21].

Complete genomes were aligned using the MAFFT software v7 [22]. Conserved motifs were aligned using the MUSCLE algorithm [23], executed within the UGENE platform [24]. The RdRp ORF was predicted using the online tool ORFfinder v0.4.3 (accessed on September 12, 2025) [25]. The prediction of RdRp domains and motifs was performed using the Motif Finder (https://www.genome.jp/tools/motif, accessed on September 17, 2025) [26]. Conserved Domains Tool v0.4.4 [27] and InterPro [https://www.ebi.ac.uk/interpro/search/sequence, accessed on August 12, 2025] [28]. To validate the discovery of new viruses, the LucaProt server ([https://lucaprot.org, accessed on September 18, 2025) [29] and PalmAnnot were used [30].

Furthermore, the multiple alignment of RdRp sequences reveals the conservation of catalytic motifs A, B, and C, which are characteristic of the *Mononeg_RNA_pol* domain. These motifs contain functionally essential residues, including Aspartate (D), Lysine (K), and Tryptophan (W), distributed across different regions of the catalytic core.

Three-dimensional modeling of viral proteins was carried out in Swiss-Model [31], and the resulting structures were visualized and annotated in UCSF Chimera [32], allowing the spatial identification of conserved catalytic sites and comparison across taxa. Phylogenetic analyses based on protein sequences supported the evolutionary placement of the novel viruses within *Lispiviridae*.

Additionally, phylogenetic analysis based on protein sequences grouped the viruses into distinct clades, highlighting evolutionary relationships and functional homology. The integration of these approaches not only enabled functional inference of the proteins but also supported the proposal of novel viral taxa, reinforced by low sequence identity with previously described viruses and the preservation of key structural and catalytic elements.

### Structural modeling of RdRp proteins

Amino acid sequences of the RdRp of the WcLispV-SP, proteins obtained from the detected viruses were modeled using Swiss-Model (https://www.swissmodel.expasy.org/interactive, accessed October 14, 2025) [31]. Visualization of the tridimensional conformation and localization of conserved motifs (A, B, and C) was performed using UCSF Chimera v1.17 (https://www.cgl.ucsf.edu/chimera). The rendering process followed the guidelines described by Pettersen et al. (2004) [32].

### Genetic nucleotide identity

Pairwise genetic distances and standard errors were computed using the Maximum Likelihood approach with 1000 bootstrap replicates. Sequence similarity matrices were generated with SDT software [33], employing the MUSCLE algorithm for pairwise alignment [23]. A phylogenetic tree based on identity scores was generated using the NEIGHBOR module from the PHYLIP package, applying the Neighbor-Joining method to depict evolutionary relationships graphically.

### Phylogenetic inference

Phylogenetic trees were inferred using the maximum likelihood (ML) method implemented [34] in IQ-TREE v1.6.12 [35], with 1000 bootstrap replicates for node support [36]. Tree visualization and annotation were conducted using FigTree v1.4.2 [37]. The sequencing data generated in this study have been deposited in the NCBI Sequence Read Archive (SRA) under the accession numbers BioProject (PRJNA1450831), BioSample (SAMN57172359), and SRA (SRR38011347), ensuring public access to the raw data. In addition, the viral sequence obtained (WcLispV-SP) was submitted to GenBank and is available under the accession number PX992751.

## Results

### Taxonomic classification

**Fig. 1:**
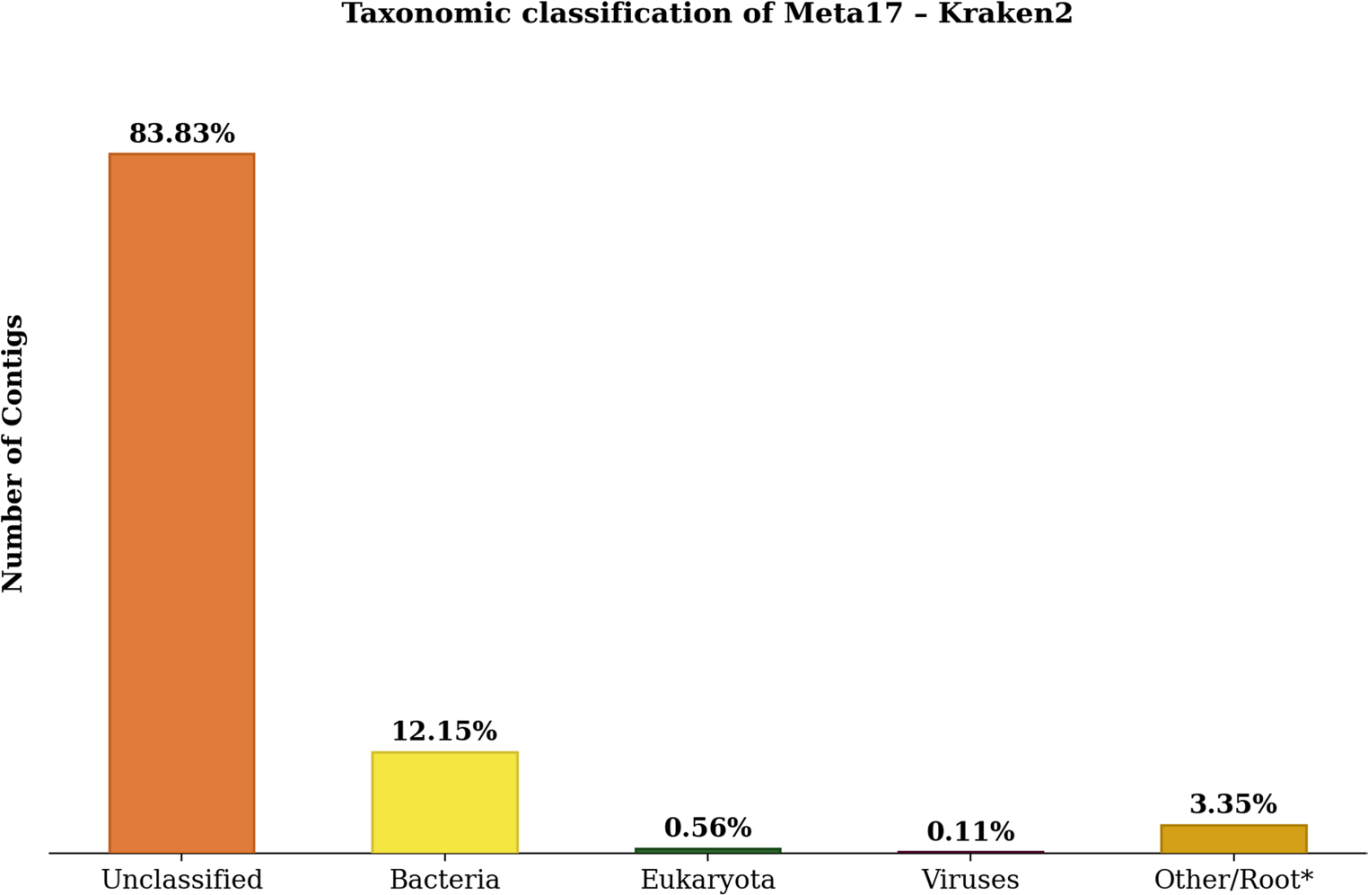
Taxonomic classification of Meta17 library. For taxonomic classification, we used Kraken2 configured with the prebuilt RefSeq Standard-Full database, which encompasses sequences from archaea, bacteria, viruses, plasmids, the human genome, and UniVec_Core

The NGS sequencing of the Meta17 library resulted in 897 contigs after *de novo* assembly. Initial taxonomic classification, performed using the Kraken2 software, revealed a predominance of unclassified contigs (83.83%), followed by Bacteria (12.15%), Eukaryota (0.56%), Viruses (0.11%), and others (3.35%), as illustrated in Fig. 1.

Within the viral fraction detected by Kraken2 (0.11%), sequences were mostly assigned to DNA viruses from the kingdom *Duplodnaviria*, including representatives of *Heunggongvirae*, *Uroviricota*, and *Caudoviricetes*, as well as a contig related to the bacteriophage *Bacteroides phage B124-14*.

The analysis conducted with DIAMOND, using the RdRp database from the *Serratus* project, showed specificity for RNA viruses. This step identified three contigs (*n* = 3), each corresponding to approximately 0.11% of the total library. Within the kingdom *Riboviria*, these sequences were distributed into three distinct categories: a double-stranded RNA virus (dsRNA viruses), a member of the family *Lispiviridae*, and an unassigned viral sequence (not assigned).

The contig designated as “not assigned” (873 nt) showed no significant similarity to sequences deposited in public databases and, therefore, was not further characterized. The dsRNA sequence was identified as *WcOrthotV-SP* (GenBank: PZ285839), belonging to the family *Orthototiviridae* and the genus *Totivirus*, the detailed description of which will be presented in a timely study. Given the higher taxonomic resolution, genomic completeness, and phylogenetic relevance of the contig associated with the family *Lispiviridae*, the present work focuses exclusively on the detailed characterization of this viral lineage.

### Genomic characterization of *Wyeomyia confusa Lispivirus* (WcLispV-SP)

Following its taxonomic placement, the genomic features of WcLispV-SP were further characterized. The genome was considered a putative complete genome based on the presence of all expected open reading frames (ORFs) characteristic of the *Lispiviridae* family, conserved genome organization, and the absence of sequence gaps across the assembled contig.

The genome of WcLispV-SP (GenBank accession number PX992751) consists of a negative-sense single-stranded RNA (ssRNA−) genome totaling 12,698 nucleotides. To further support genome completeness, read mapping analysis using Bowtie2 (very-sensitive end-to-end mode) and Qualimap revealed a mean sequencing depth of 33.96× (SD = 19.62) across the genome, with a total of 431,184 mapped bases. Despite some variability in coverage, these results indicate sufficient sequencing depth to support the reliability of the assembled genome, consistent with patterns commonly observed in metagenomic datasets.

**Fig. 2:**
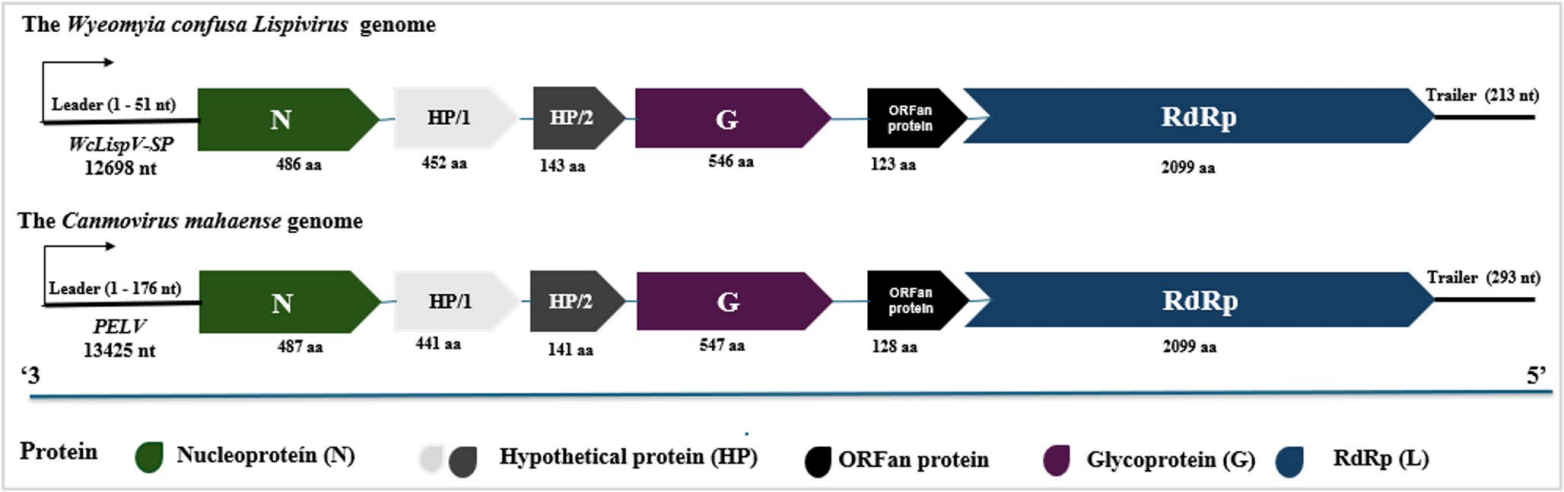
Genomic organization of *Wyeomyia confusa* Lispivirus (WcLispV-SP), composed of 12,698 bp of negative-sense single-stranded RNA (ssRNA⁻). The genome encodes six major ORFs: N (nucleoprotein, green), HP/1 and HP/2 (hypothetical proteins of unknown function, light and dark gray), G (glycoprotein, purple), ORFan protein (black), and RdRp (RNA-dependent RNA polymerase, dark blue). Colors represent the functional identity of each coding region. The overall genomic architecture is consistent with viruses belonging to the family *Lispiviridae*. A comparative genomic organization with *Canmovirus mahaense* (PELV) is also shown

Comparative analysis using BLASTn revealed 81.12% identity and 50% coverage with Pedras Lispivirus (OQ779241.1), previously identified in *Sabethes quasicyaneus* in Maranhão State, Brazil [37]. The viral genome encodes six open reading frames (ORFs): nucleoprotein (N), two proteins of unknown function (HP/1 and HP/2), glycoprotein (G), ORFan protein, and RNA-dependent RNA polymerase (RdRp-L) (Table 1). The genomic organization of WcLispV-SP shows the arrangement of the six ORFs along the viral genome (Fig. 2). The N gene is positioned at the 3′ end, followed by HP/1 and HP/2. The G gene is located downstream, and the RdRp-L gene occupies the 5′ terminus, consistent with the structural organization observed in members of the *Lispiviridae* family.

**Table 1:** Genomic features of *Wyeomyia confusa* Lispivirus (WcLispV-SP) compared with its best-hit homolog (PELV) in GenBank

Genomic features	Length (nt)		Coverage (%)		E-values		Identity (%)	
	WcLispV-SP	Canmo	WcLispV-SP	Canmo	WcLispV-SP	Canmo	WcLispV-SP	Canmo
_PELV		_PELV		_PELV		_PELV		_PELV
Genome	12,698	13,425	50%	100%	0 × 10^0^	0 × 10^0^	81.12%	100%
RdRp	2099	2099	100%	100%	0 × 10^0^	0 × 10^0^	81.12%	100%
ORFan protein	123	128	–	–	–	–	–	–
Putative glycoprotein (G)	547	546	99%	100%	1 × 10^− 83^	0 × 10^0^	66.79%	100%
Nucleoprotein (N)	486	487	100%	100%	0 × 10^0^	0 × 10^0^	81.69%	100%
Hypothetical protein (HP/1)	452	441	98%	100%	1 × 10^− 83^	0 × 10^0^	36.20%	100%
Hypothetical protein (HP/2)	143	141	97%	100%	1 × 10^− 64^	2 × 10^− 99^	86.47%	100%

(–) No detectable similarity in available databases

At the genomic termini, WcLispV-SP contains a predicted 51 nt leader region at the 3′ end and a predicted 213 nt trailer region at the 5′ end, in accordance with genomic features described for members of the order *Mononegavirales*. These terminal non-coding regions are known to play important roles in transcription initiation, replication, and genome encapsidation, further supporting the classification of WcLispV-SP within the *Lispiviridae* family. However, experimental validation of the genomic termini (e.g., RACE) was not performed. Comparison between WcLispV-SP and *Pedras Lispivirus* revealed high levels of similarity, although with significant variation across different ORFs. The proteins corresponding to the glycoprotein (G, 547 aa; GenBank: WHM52804.1), protein HP/1 (452 aa; GenBank: WHM52802.1), ORFan protein (123 aa) no detectable similarity in available databases and RdRp-L (2099 aa; GenBank: WHM52805.1) showed coverage of 99%, 98%, and 100%, with sequence identities of 66.79%, 36.20%, and 81.12%, respectively, in relation to the virus identified in *Sabethes quasicyaneus* [38]. In contrast, the proteins corresponding to the nucleoprotein (N, 486 aa; GenBank: XGU11753.1) and protein HP/2 (143 aa; GenBank: XGU11754.1) exhibited coverage of 100% and 97%, with sequence identities of 81.69% and 86.47%, respectively, when compared to *Pedras Lispivirus* isolated from *Sabethes albiprivus* in the state of Minas Gerais, Brazil, also in 2021 [39]. 

These findings indicate that WcLispV-SP represents a novel viral species within the *Lispiviridae* family, possibly associated with wild mosquito species in the Neotropical region, contributing to the understanding of viral diversity and evolution in Brazilian ecosystems.

### Analysis of Domains and Conserved Motifs of the *Lispiviridae *Family

**Fig. 3:**
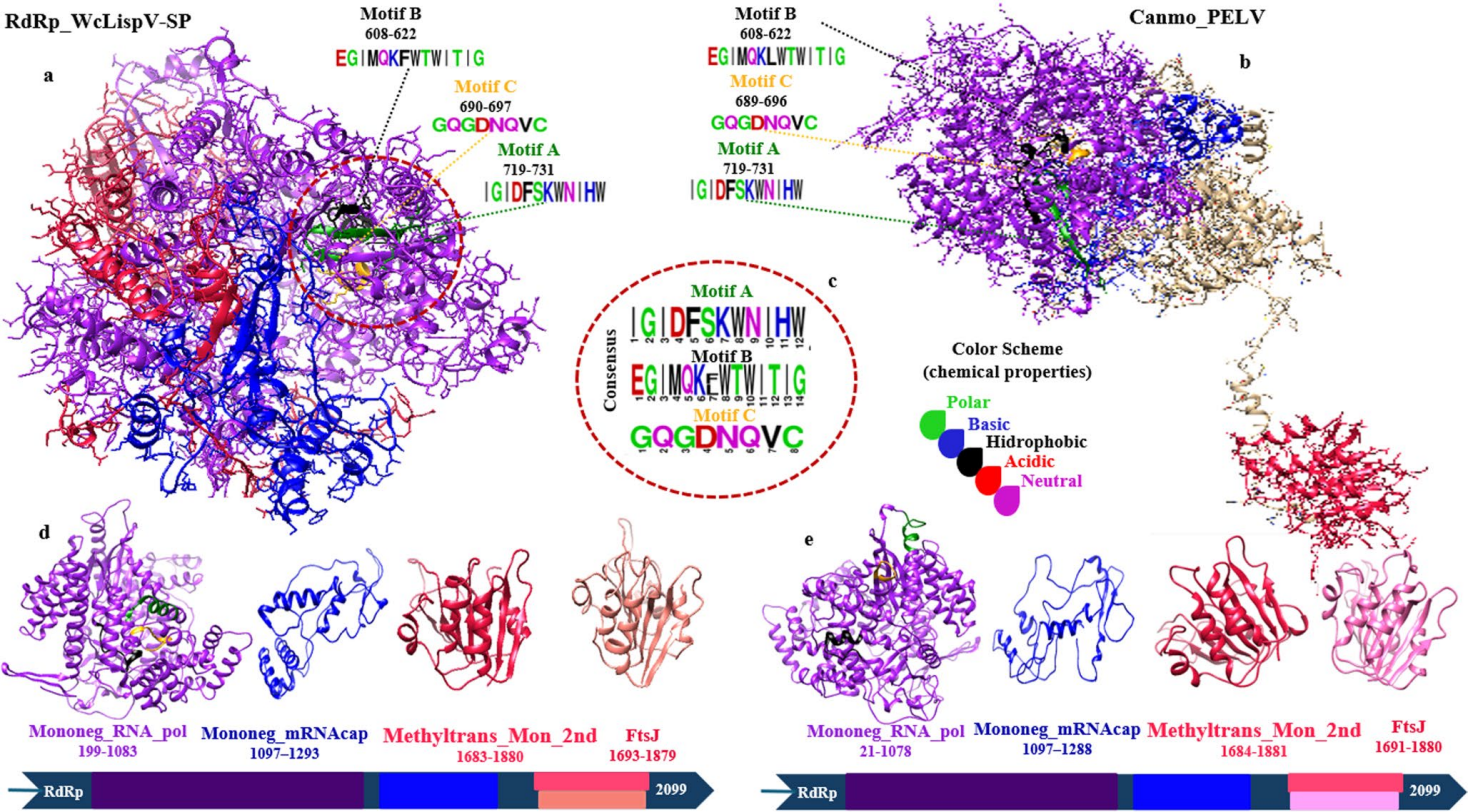
Comparative structural representation of RdRp proteins from WcLispV-SP (**a**) and Canmo_PELV (**b**), highlighting conserved catalytic motifs (A, B, and C) and the organization of functional domains. (**a**) Three-dimensional structure of RdRp_WcLispV-SP (2099 amino acids), showing motifs A (719–731), B (608–622), and C (690–697) located within the *Mononeg_RNA_pol* domain (199–1083). (**b**) Corresponding structure of RdRp_Canmo_PELV (2099 amino acids), demonstrating topological conservation of the same catalytic motifs (A: 719–731; B: 605–622; C: 695–696). Motif B (sequence: EGIMQKFWTWITIG; position: 608–622; 14 amino acids) of WcLispV-SP RdRp shows high similarity to the corresponding sequence in Canmo_PELV, differing only at position 7, where phenylalanine (F) is replaced by leucine (L) (EGIMQKLWTWITIG). (**c**) Sequence logo of motifs A, B, and C, color-coded by the chemical properties of residues: green (polar), blue (basic), black (hydrophobic), red (acidic), and pink (neutral). d-e. Modular representation of the domains in RdRp_WcLispV-SP and RdRp_Canmo_PELV, respectively: *Mononeg_RNA_pol*, *Mononeg_mRNAcap*, *Methyltrans_Mon_2nd*, and *FtsJ*, with indication of amino acid intervals. Structural conservation between both polymerases suggests functional similarity among viral species of the *Lispiviridae* family, preserving the catalytic and methyltransferase domains typical of viruses in the *Mononegavirales* order

Structural and comparative characterization of the RNA-dependent RNA polymerase (RdRp) sequence of the WcLispV-SP virus, associated with the mosquito *Wyeomyia confusa*, revealed the presence of four conserved domains. These include: the *Mononegavirales* RNA-dependent RNA polymerase domain (*Mononeg_RNA_pol*, PF00946), which encompasses structural motifs A, B, and C; the *Mononegavirales* mRNA-capping region V domain (*Mononeg_mRNAcap*, PF14318); the virus-capping methyltransferase domain (*Methyltrans_Mon_2nd*); and the *FtsJ-like methyltransferase* domain (*FtsJ*, PF01728) (Fig. 3; Table 2).

**Table 2:** Comparative identification of conserved domains in the RNA-dependent RNA polymerase (RdRp) of *Wyeomyia confusa lispivirus* (WcLispV-SP) and Canmo_PELV

Protein	Pfam ID	Pfam domain	Position (aminoacids)		i-Evalue	
			WcLispV-SP	Canmo_PELV	WcLispV-SP	Canmo_PELV
RdRp	PF00946	*Mononeg_RNA_pol*	0199–1083	0021-1078	2.8 × 10^− 129^	1.3 × 10^− 133^
	PF14318	*Mononeg_mRNAcap*	1097–1293	1097–1288	9.4 × 10^− 34^	3.4 × 10^− 34^
	PF14314	Methyltrans_Mon_2nd	1683–1880	1684–1881	2.8 × 10^− 07^	7.2 × 10^− 7^
	PF01728	FtsJ	1693–1879	1691–1880	12.8 × 10^− 4^	1.9 × 10^− 1^

Comparison of the RdRp proteins from WcLispV-SP and Canmo_PELV revealed four conserved domains typical of the order *Mononegavirales*. Positions correspond to amino acid residues within each RdRp sequence. The identified domains include: *Mononegavirales* RNA-dependent RNA polymerase (*Mononeg_RNA_pol*), *Mononegavirales* mRNA-capping region V (*Mononeg_mRNAcap*), Virus-capping methyltransferase MT domain (Methyltrans_Mon_2nd), and FtsJ-like methyltransferase (FtsJ). The i-Evalue represents the statistical significance of each domain alignment obtained from Pfam analysis.

Three-dimensional modeling of RdRp (WcLispV-SP) was performed using SWISS-MODEL, based on multiple L protein templates from *Mononegavirales* (main templates: 8kdb, 8jsm, 6u1x, 9oce, among others). The templates covered most of the L ORF (approximate range: 146–1959; coverage ~ 0.80–0.86), although sequence identity between the target and the templates was low (~ 16–20%), resulting in moderate global quality estimates (GMQE ~ 0.26–0.39; QMEANDisCo global ≈ 0.45–0.51 ± 0.05) [27].

Consequently, the model is reliable for inferring the global topology and relative positioning of catalytic motifs A, B, and C, but should not be used to draw conclusions about detailed atomic interactions (e.g., precise coordination of metal ions) without further validation. Ligands and metal ions present in the templates were not transferred, as the binding sites were not conserved in the alignments.

Additionally, three-dimensional modeling of the RdRp from Canmo_PELV was also performed using SWISS-MODEL under the same conditions described above, based on multiple L protein templates from *Mononegavirales* (coverage ~ 0.78–0.82; GMQE 0.32–0.37; QMEANDisCo ≈ 0.48–0.52) [27].

In addition to domain identification, the three-dimensional structural analysis of the protein (Fig. 3) reveals the modular arrangement typical of polymerases from the *Mononegavirales* order. The *Mononeg_RNA_pol* domain, located between residues 199–1083, constitutes the catalytic core of the enzyme and contains motifs A, B, and C, which are responsible for binding to the RNA template and coordinating the metal ions required for nucleotide catalysis. Following this, the *Mononeg_mRNAcap* domain (1097–1293) forms a functional subunit associated with the initiation of mRNA capping, participating in the transfer of guanosine triphosphate (GTP) to the 5′ end of the nascent transcript [40].

In the C-terminal region, the *Methyltrans_Mon_2nd* domain (1683–1880) and the *FtsJ-like methyltransferase* domain (1693–1879) act in a coordinated manner to methylate the cap structure, promoting sequential modifications on the guanosine and the 2′-O ribose residues. This modular organization, from the polymerase core to the methyltransferase domains, reflects the multifunctional arrangement observed in other RdRps from *Mononegavirales*, such as those of *Vesiculovirus* and *Paramyxovirus* [40, 41].

In an integrated manner, the domains of RdRp_WcLispV-SP enable the same protein to catalyze RNA synthesis, cap addition and methylation, and post-transcriptional processing, ensuring replicative efficiency and viral RNA stability. This architecture reinforces structural conservation among members of the *Mononegavirales* order, even in phylogenetically distinct viruses such as those in the *Lispiviridae* family.

Given the functional relevance of the *Mononeg_RNA_pol* domain, a structural analysis was conducted on the conserved motifs A, B, and C, which constitute the catalytic core of RdRp. The three-dimensional model of the protein shows that these motifs are organized within the catalytic palm region, displaying a spatial arrangement similar to that observed in the RdRp of *Pedras Lispivirus* (WHM52805.1).

Motif A (sequence: IGIDFSKWNIHW; position: 719–731; 12 amino acids), located within the *Mononeg_RNA_pol* domain of WcLispV-SP RdRp, is identical to motif A found in the truncated sequence of the Canmo_PELV protein, differing only in the residue interval (range: 21–1078).

Comparative analysis of 53 motifs A sequences among members of the *Lispiviridae* family revealed a highly conserved amino acid pattern (xxxDxxxWNxxx), located in a structurally essential region of the enzyme, suggesting a critical functional role in catalysis (Fig. 3c). The tryptophan residue (W) contributes to stabilizing the interface between the polymerase and the RNA template, facilitating proper alignment during transcription, while the aspartate residue (D) is involved in coordinating metal ions and positioning nucleotides during synthesis.

Asparagine (N), in turn, can establish hydrogen bond interactions with the RNA or with other residues of the enzyme, helping to maintain the architecture of the active site and promoting precise nucleotide recognition. The remaining residues that compose motif A directly influence elongation efficiency, contributing to conformational stability and sustaining the catalytic activity of the enzyme.

Motif B (sequence: EGIMQKFWTWITIG; position: 608–622; 14 amino acids) of WcLispV-SP RdRp shows high similarity to the corresponding sequence in the Canmo_PELV protein, differing only at position 7, where phenylalanine (F) is substituted by leucine (L) (EGIMQKLWTWITIG). This point variation, located in a structurally conserved region, does not alter the overall conformation of the motif, indicating preservation of its essential catalytic function.

Among members of the *Lispiviridae* family, comparative analysis revealed that motif B follows a consensus pattern (xGxxQKxWT, *n* = 5), positioned in the central region of the *Mononeg_RNA_pol* domain, adjacent to motif A. Structurally, this motif contributes to the alignment and stabilization of the RNA template strand, functioning as a coupling element between the active site and the RNA entry channel within the catalytic cavity.

Structural evidence from studies of homologous polymerases suggests that the residues Q (glutamine), K (lysine), and W (tryptophan) play crucial roles in RNA interaction and in maintaining the architecture of the active site, promoting correct template positioning during elongation.

Glycine (G), due to its small side chain, provides conformational flexibility to the local structure, allowing dynamic adjustment between the palm domain and neighboring motifs during catalysis. Threonine (T), in turn, contributes to the stabilization of hydrogen bonds and to the positioning of nucleotides within the catalytic channel, cooperating with the charged residues of motif C during the incorporation process.

Thus, motif B likely functions as a regulatory module for the fidelity and catalytic efficiency of RdRp, playing an important role in the conformational stability of the enzymatic complex during the replication process.

**Fig. 4:**
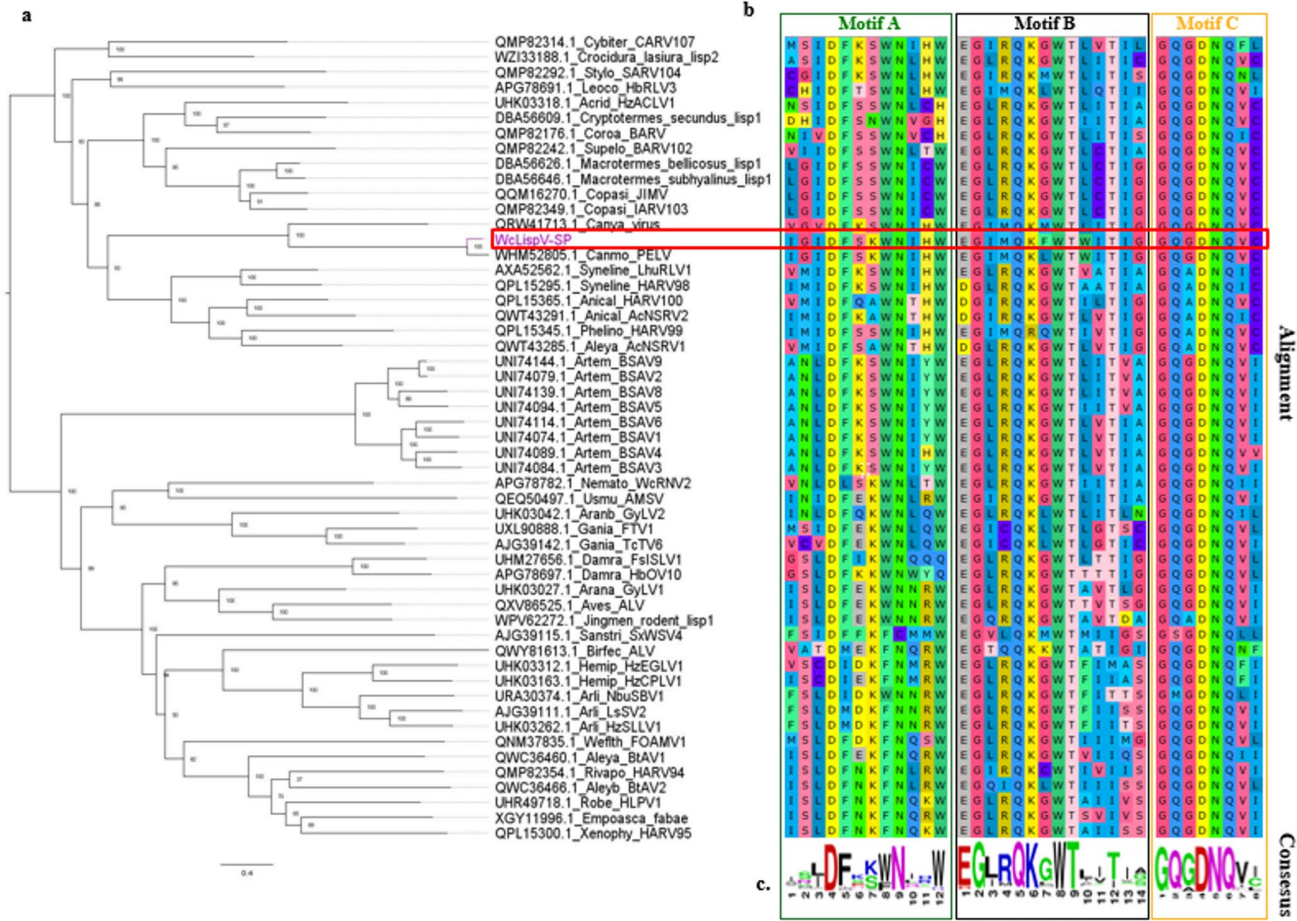
Phylogenetic analysis and conservation of catalytic motifs in the RdRp protein of *Wyeomyia confusa Lispivirus* (WcLispV-SP). (a) Phylogenetic tree inferred from 53 RNA-dependent RNA polymerase (RdRp) protein sequences representing classified and unclassified species of the *Lispiviridae* family. WcLispV-SP clusters in a monophyletic clade with *Pedras Lispivirus* (Canmo_PELV), supported by a high bootstrap value (100), indicating a close evolutionary relationship between the two species. (b) Multiple alignment of conserved regions of RdRp highlighting catalytic motifs A, B, and C, typical of the *Mononeg_RNA_pol* domain. The conservation of these residues among *Lispiviridae* representatives demonstrates the functional preservation of catalytic sites associated with polymerase activity. (c) Consensus amino acid sequence

Motif C (sequence: GQGDNQVC; position: 690–697, 8 amino acids) of WcLispV-SP RdRp is identical to the corresponding sequence in the Canmo_PELV protein. Among the 53 sequences analyzed from the *Lispiviridae* family, the motif displays the conserved pattern GxxDNQxx, characteristic of the catalytic “palm” domain of viral polymerases (Figs. 3c and 4c). This region directly participates in the coordination of divalent metal ions, such as Mg²⁺ or Mn²⁺, which are essential for nucleotide catalysis. The glycine (G) residue, due to its high flexibility, contributes to structural adjustment of the active site, allowing proper positioning of the RNA template.

The aspartate residue (D), due to its acidic nature, plays a central role in catalysis by participating in ribonucleotide activation and stabilizing the enzymatic complex. Asparagine (N) and glutamine (Q), both polar residues, promote the formation of hydrogen bonds and interactions with RNA or adjacent residues, contributing to structural stability and precision during the polymerization process. The presence of motifs A (xxxDxxxWNxxx), B (xGxxQKxWT, *n* = 5), and C (GxxDNQxx), which are characteristic of the L protein in viruses of the *Lispiviridae* family, reinforces the multifunctionality of this enzyme and the structural conservation across the different sequences analyzed [40, 42–45].

### Phylogenetic analysis

The phylogenetic tree was inferred from 53 amino acid sequences of RdRp proteins, including 45 classified sequences and 8 not yet recognized by ICTV. Phylogenetic inference shows that WcLispV-SP forms a monophyletic clade with *Pedras Lispivirus* (Canmo_PELV), supported by a high bootstrap value (1000) and the application of the LG + F+R8 substitution model, confirming their close evolutionary relationship. This relationship is consistent with the results obtained using the Species Demarcation Tool (SDTv), which indicated 81.1% similarity between the two proteins. This value falls within the demarcation thresholds defined by ICTV for the *Lispiviridae* family, in which distinct species within the same genus share less than 85% amino acid identity (Fig. 4).

Multiple sequence alignment of RdRp proteins reveals high conservation of catalytic motifs A, B, and C, hallmarks of the *Mononeg_RNA_pol* domain, among representatives of the *Lispiviridae* family. This structural conservation reflects the preservation of the essential catalytic mechanism and reinforces the evolutionary relationship between WcLispV-SP and other members of the *Canmovirus* genus.

BLASTp analysis of the RdRp sequence from WcLispV-SP revealed 100% coverage and 81.12% identity with the RdRp of *Pedras Lispivirus* (WHM52805.1), previously identified in *Sabethes quasicyaneus*. This additional similarity corroborates the results obtained through SDTv, reinforcing the phylogenetic clustering and genomic proximity between both viruses. Taken together, the phylogenetic results, identity indices, and conservation of catalytic motifs support that WcLispV-SP represents a novel viral species within the *Canmovirus* genus, in accordance with the taxonomic criteria established by ICTV [21, 40].

## Discussion

The characterization of *Wyeomyia confusa Lispivirus* (WcLispV-SP) broadens current understanding of viral diversity associated with Neotropical sylvatic mosquitoes, particularly within the Atlantic Forest biome, one of the most biodiverse regions worldwide [46]. The detection of this virus in *W. confusa* highlights the relevance of metagenomic approaches for uncovering highly divergent viral lineages that are often missed by conventional surveillance strategies. The phylogenetic proximity between WcLispV-SP and *Pedras Lispivirus*, previously identified in *Sabethes quasicyaneus* and *Sabethes albiprivus*, suggests that members of the *Lispiviridae* family may be widely distributed among strictly sylvatic mosquitoes occupying comparable ecological niches across different regions of Brazil [39].

A key limitation of this study is the use of a single metagenomic pool comprising 44 *W. confusa* specimens. Although pooling is commonly applied in mosquito virome studies, it constrains the ability to estimate true viral prevalence, assess intra-species variability, and determine individual-level infection patterns. Previous reports indicate that pooled analyses may underestimate viral diversity or obscure coinfections, particularly when viruses are present at low abundance [47]. Additionally, the absence of biological replicates prevents statistical evaluation of detection consistency across distinct sampling events or locations. Therefore, these findings should be interpreted as initial evidence of the presence of a novel lispivirus in *W. confusa*, rather than a population-level estimate.

Despite the implementation of stringent bioinformatic pipelines, metagenomic approaches remain susceptible to environmental or laboratory cross-contamination. The recovery of a single contig with uniform genomic coverage reduces the likelihood of assembly artifacts, but does not completely exclude this possibility. Recent studies emphasize that low-level contaminants may generate false-positive signals, especially for highly divergent or poorly represented viruses in reference databases [47]. Furthermore, the lack of experimental validation, such as PCR or RACE, limits confirmation of genomic termini. While the available data strongly support the authenticity of the assembled genome, additional experimental validation would strengthen these findings.

The detection of WcLispV-SP exclusively in *W. confusa* does not support conclusions regarding strict host specificity. Viruses within the *Lispiviridae* family have been reported in multiple genera of Neotropical mosquitoes, including *Sabethes* and *Haemagogus*, indicating potential ecological flexibility and the capacity to infect different hosts [48]. The phylogenetic relatedness between WcLispV-SP and *Pedras Lispivirus* is consistent with this interpretation. Moreover, the predominantly zoophilic behavior of *W. confusa* raises the possibility of indirect exposure to sylvatic vertebrates, although there is currently no evidence that lispiviruses infect vertebrate hosts. Future studies incorporating broader metagenomic sampling and experimental assessments of cell tropism will be essential to clarify host range.

The occurrence of WcLispV-SP supports the view that Neotropical ecosystems harbor extensive and still underexplored viral diversity. The detection of related lispiviruses in different Brazilian states, including Maranhão, Minas Gerais, and São Paulo, suggests a broad geographic distribution associated with forested and ecologically similar environments [39]. Similar distribution patterns have been described for other viruses linked to sylvatic mosquitoes, such as members of the genera *Phlebovirus* and *Orthobunyavirus* [49, 50]. These observations highlight the importance of surveillance efforts in biodiverse regions, where complex ecological interactions contribute to the maintenance and diversification of viral communities.

Recent virome investigations in Brazilian mosquitoes have revealed a wide array of highly divergent RNA viruses, many of which remain taxonomically unclassified [51]. These findings place WcLispV-SP within a broader and still expanding landscape of mosquito-associated viral diversity. In particular, they reinforce the role of strictly sylvatic genera such as *Wyeomyia* and *Sabethes* as important reservoirs of largely uncharacterized viruses, underscoring the need for continued metagenomic exploration.

In this broader context, members of the *Lispiviridae* family exhibit considerable genomic and ecological diversity, with negative-sense single-stranded RNA genomes ranging from approximately 6.5 to 15.5 kb and encoding multiple structural and non-structural proteins, including the RNA-dependent RNA polymerase (RdRp) [24]. Comparative analysis of the RdRp of WcLispV-SP revealed conservation of key catalytic motifs and domain architecture consistent with viruses of the order *Mononegavirales* [41]. Minor amino acid substitutions observed in conserved regions may reflect adaptive processes without necessarily affecting enzymatic function, although experimental validation is required to assess their functional impact. These features emphasize the evolutionary stability of core replication machinery alongside the potential for fine-scale adaptive variation.

Currently, the *Lispiviridae* family comprises multiple genera and species infecting a broad spectrum of invertebrate hosts, including insects, nematodes, and other arthropods of ecological and economic relevance [21, 52–57]. This wide host distribution highlights the adaptive versatility of these viruses and suggests long-term coevolutionary relationships with diverse hosts across different ecological niches.

Although there is no evidence that lispiviruses infect humans or other vertebrates, the identification of novel viruses in sylvatic mosquitoes contributes to understanding long-term evolutionary processes that may influence viral emergence. Historical examples of arboviruses, including Zika, chikungunya, and yellow fever viruses, demonstrate that pathogens previously restricted to sylvatic cycles can expand their host range under specific ecological conditions [58]. In this context, the characterization of WcLispV-SP provides relevant insights into the evolutionary dynamics shaping virus–host interactions in Neotropical environments.

In summary, the identification of WcLispV-SP in *Wyeomyia confusa* reinforces the importance of metagenomic surveillance in sylvatic mosquito populations and contributes to refining current knowledge of the diversity, evolution, and host associations of the *Lispiviridae* family. Continued investigation of such viruses is essential for advancing our understanding of RNA virus evolution and the ecological and evolutionary processes that shape their distribution in natural environments.

## Conclusion

In this study we have performed a genomic characterization of a novel virus associated with the mosquito *Wyeomyia confusa* (WcLispV-SP), detected in samples collected from the Atlantic Forest in Pindamonhangaba, São Paulo, Brazil. Phylogenetic analyses and distance-based metrics indicate that WcLispV-SP represents a new species within the genus *Canmovirus*, belonging to the *Lispiviridae* family, according to the demarcation criteria established by ICTV. These results show that metagenomics has the potential to be an effective tool for viral surveillance and discovery, able to identify lineages that silently move around in environments with high biodiversity. In order to forecast possible spillover risks and comprehend the evolutionary dynamics of viruses in arthropods, more research is necessary. This will help develop integrated strategies for genomic surveillance and environmental monitoring within the context of public health and the One Health approach.

## Data Availability

The sequencing data generated in this study are publicly available in the NCBI Sequence Read Archive (SRA) under the following identifiers: BioProject PRJNA1450831, BioSample SAMN57172359, and SRA SRR38011347; as well as the viral sequence characterized in this study, which has been deposited in GenBank under accession number PX992751 (WcLispV-SP).
